# Spatiotemporal Dynamics of Multiple Memory Systems During Category Learning

**DOI:** 10.3390/brainsci10040224

**Published:** 2020-04-09

**Authors:** Kyle K. Morgan, Dagmar Zeithamova, Phan Luu, Don Tucker

**Affiliations:** 1 Brain Electrophysiology Laboratory Company, Eugene, OR 97403, USA; phan.luu@belco.tech (P.L.); don.tucker@belco.tech (D.T.); 2 Department of Psychology, University of Oregon, Eugene, OR 97403, USA; dasa@uoregon.edu

**Keywords:** category learning, eeg, machine learning, erp, memory, learning, multiple memory systems, p300

## Abstract

The brain utilizes distinct neural mechanisms that ease the transition through different stages of learning. Furthermore, evidence from category learning has shown that dissociable memory systems are engaged, depending on the structure of a task. This can even hold true for tasks that are very similar to each other, which complicates the process of classifying brain activity as relating to changes that are associated with learning or reflecting the engagement of a memory system suited for the task. The primary goals of these studies were to characterize the mechanisms that are associated with category learning and understand the extent to which different memory systems are recruited within a single task. Two studies providing spatial and temporal distinctions between learning-related changes in the brain and category-dependent memory systems are presented. The results from these experiments support the notion that exemplar memorization, rule-based, and perceptual similarity-based categorization are flexibly recruited in order to optimize performance during a single task. We conclude that these three methods, along with the memory systems they rely on, aid in the development of expertise, but their engagement might depend on the level of familiarity with a category.

## 1. Introduction

Category learning has been a productive paradigm for studying learning and memory and it refers to the development of the ability to group objects belonging to the same category and differentiate objects belonging to different categories [1]. There is not a single mechanism of category learning. Research using category learning models have outlined that there are distinct neural mechanisms associated with different learning stages [2]. Furthermore, we know that different tasks engage dissociable memory systems that are optimized for the type of learning involved—even for seemingly similar tasks, such as in categorization [3,4,5,6,7]. This makes it difficult to uniquely attribute the changes in brain activity to either distinct learning systems or representations of the distinct mechanisms that are associated with different task sets. In other words, the two bodies of literature can complicate comparing brain activity across tasks as subjects between those tasks could either be at different stages of learning or be relying on different categorization strategies that are served by dissociable memory systems.

From the skill acquisition perspective, a succinct model of learning has been proposed that describes reliance upon executive functions, depending on the stage of learning: early or late. Under the dual-stage model, the early stage of learning is marked by being heavily reliant on controlled processes, requiring a person to be actively attentive and dependent on limited working memory capacity. In contrast, the late stage is defined by its lack of reliance on controlled processes, reflected as automated performance, and it is not limited by working memory capacity and can be subconsciously carried out under the right context [2].

Modern imaging evidence has delineated distinct brain networks that are involved in the two learning stages [8]. The frontal lobe is responsible for the executive monitoring of unfamiliar stimuli; a process that is integral to the early stages of learning. In contrast, cortical regions of the posterior corticolimbic system are engaged when subjects demonstrate proficient performance in the late stages of learning [8,9]. These posterior corticolimbic structures, which include the hippocampus and posterior cingulate cortex (PCC), consolidate information and, with sufficient practice, enable performance to be more automated, thus removing the need for executive control.

Finer details regarding how the brain changes as a person learns to recognize category structures can be understood from the perspective of the dual stage theory [10]. The dual model of sensory information processing is based on evidence that suggests two separable and parallel systems operate on incoming sensory data. The first is a ventral “what” system that is responsible for the identification of an event or object and it includes the sensory specific cortices (such as visual cortex) and the ventral limbic system, which includes the parahippocampal gyrus, piriform, entorhinal cortex, and the amygdala [11,12]. The second processing stream, as exemplified by dorsal cortical regions of the parietal lobe, is referred to as the dorsal or “where” pathway, and it specializes in spatial analysis of stimuli [11,12]. Information from both streams converge at the hippocampus, which is a structure situated in the medial temporal lobe (MTL) that plays a key role in organizing input to link memories by their contextual representation [13]. This feedback structure allows for the hippocampus to organize memory retrieval based off “what” occurred or “where” something occurred and makes it an essential mechanism for memory retrieval. With further training, the hippocampus is able to perform declarative recall with less need for controlled attention and input from these two sensory pathways; reflecting the early/late transition outlined in the dual stage model. However, a relevant shortcoming to both the dual processing and dual stage models is that as they exist, they do not account for the evidence of other types of memory systems and their possible differential reliance on the brain mechanisms that are described in the models. Another shortcoming is that they do not consider the possibility that different memory systems could be simultaneously engaged during a task, either in competition or working in conjunction, and at different learning stages to optimize learning.

Multiple mechanisms that rely on dissociable memory systems have been implicated in categorization and category learning. One distinction is between strategies that require the application of an explicit rule (rule-based categorization) and those that rely on perceptual similarity (examples: [14,15]). For example, in a family resemblance structure, stimuli that belong to the same category share several common features, with none of them being necessary or sufficient for category membership [16,17]. Categorization relies on the overall similarity rather than an explicit rule. The perceptual similarity system involves posterior visual areas and does not heavily rely on working memory [18,19,20]. Perceptual similarity allows for making rapid judgements regarding category membership without using much cognitive resources, but falls short in its ability to classify objects when within-category similarity is low or between category similarity is high [21].

In contrast, in rule-based categorization, category membership is dictated by an explicit, verbalizable rule [15]. Rule discovery is commonly achieved through explicit reasoning and hypothesis testing that heavily relies on working memory and selective attention, which are supported by the working memory system in prefrontal cortex and caudate nucleus [22]. The working memory system, within the context of rule-based categorization, allows for participants to focus on individual diagnostic dimensions while ignoring the irrelevant features within the task. This allows for accurate categorization when the within-category variance is high and between-category variance is low. However, rule-based categorization is cognitively expensive and sensitive to distractions when compared to the perceptual similarity system [3,23].

Prior research has focused on creating tasks that exaggerate the preferential recruitment of one system over another to provide compelling evidence for the existence of multiple systems. Evidence from these studies has shown that performance is hindered when the participants fail to engage the memory system optimal for a given category structure. The composition of natural categories contains elements of rule-, *and* perceptual-based systems, suggesting people may be switching between systems within a single task. Identifying signatures of distinct memory systems within single tasks would allow us to better understand how each system contributes to performance and how these systems fit within the expertise development framework.

The main goals of the presented studies were to understand the degree to which distinct learning and memory systems are recruited within the same, real-world task. We implemented a categorization task that was designed to encourage participants to switch between categorization strategies on a trial-by-trial basis and then measured the underlying neural activity in two separate experiments while using functional Magnetic Resonance Imaging (fMRI) and dense-array Electroencephalography (dEEG). The goal of our first experiment, which was a low-sample pilot, was to utilize the spatial resolution of fMRI to establish the overall effectiveness of our task in engaging different memory systems for different trials within the same task. A successful proof-of-concept and spatial distribution in the fMRI pilot then motivated the second, dEEG experiment. In our fully powered dEEG experiment, we studied the time course by which these strategies (and their underlying memory systems) were engaged on a trial-by-trial basis. More specifically, we were interested in understanding when, on a given trial, we can accurately dissociate between verbal and non-verbal rules and the associated memory systems. As the brief involvement of limited attentional and working memory resources may beneficial for the optimization of categorization strategy to the task [24], mapping the timing of the initial convergence and subsequent divergence of distinct categorization processes can provide new insights regarding how distinct systems compete and cooperate to optimize performance. Rough estimates of the anatomical differences between these systems were made while using the EEG data and a novel machine learning approach.

## 2. fMRI Pilot Experiment

### 2.1. Materials and Methods

#### 2.1.1. Participants

Eleven right-handed subjects between the ages of 18 and 30 (*M* = 24.2) were recruited from the University of Oregon Human Subjects Pool to participate in our pilot experiment (five males, six females). The subjects had no self-reported neurological or psychiatric conditions, as well as no MRI contraindications. Subjects were compensated $35 for their participation and the Electrical Geodesics, Inc. and University of Oregon IRBs approved the protocol.

#### 2.1.2. Task

**Figure 1:**
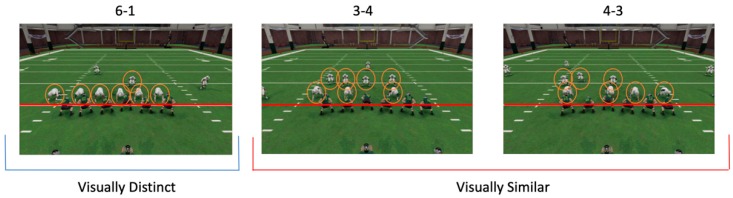
Standard formations used in the experiment. Left (blue underline): A 6-1 formation, which represents the visually distinct category, had six white players on the line of scrimmage (red line) and one player lined up behind them. Middle and Right (red underline): A 3-4 or 4-3 formation, which represent the visually similar categories, had three players (3-4) or 4 players (4-3) on the line of scrimmage (red line) and either four players (3-4) or 3 players (4-3) lined up behind them. The position of the green players did not vary significantly between formations.

The task used was designed to interchangeably recruit a rule-based or similarity-based categorization strategy to categorize three categories of football defensive formations. When between-category similarity is low and within-category similarity is high, visual similarity can guide categorization without the need of limited cognitive resources. However, when between-category similarity is relatively high compared to within-category similarity, successful categorization requires the discovery and an application of an explicit categorization rule, which taxes limited resources, such as working memory and attention. Thus, we chose two formation categories that were visually similar to each other and one category that was visually distinct from the other two. For the two similar categories, the subjects needed to discover an explicit counting rule in order to categorize the members of these two groups reliably: One category of formations displayed three people on the line of scrimmage (and four behind them, 3-4 category), while the other had four people on the line of scrimmage (and three behind them, 4-3 category) (Figure 1). Because of the variable starting positions among players, the formations that fell into one category (e.g., 3-4) could look different from each other (within-category variability). In addition, some of the formations in the 3-4 category were visually very similar to some of the formations in the 4-3 category (high between-category similarity). Thus, the participants could not easily rely on visual similarity and instead needed to discover the rule (number of people on the line of scrimmage) that differentiated the categories. This forced subjects to focus their attention to the line of scrimmage while ignoring irrelevant players positioned elsewhere on the field. We expected that, although these were two categories that participants needed to differentiate between, stimuli from both categories would evoke the same cognitive processes (explicit, rule-based categorization). We collapsed over them in neuroimaging analyses when looking for neural processes associated with these rule-based trials, as subsequent analyses confirmed that participants’ performance was comparable for the 3-4 and 4-3 categories on all metrics.

For the visually distinct category, there was also an explicit rule, with six people on the line of scrimmage and one person behind (6-1) (Figure 1). However, this category was visually sufficiently distinct from the 3-4 and 4-3 categories, so subjects could rely on visual similarity alone during categorization, rather than having to discover and invoke an explicit rule.

**Figure 2:**
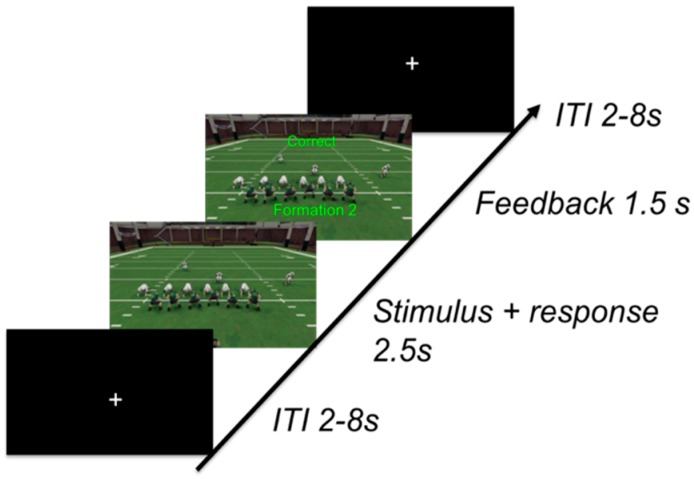
Formations were shown for 2.5 s. Immediately following a response, contingent feedback was shown for 1.5 s. Upon feedback termination, a fixation mark was shown for the duration of the inter-trial interval of 2–8 s before the next formation was presented.

Every category had three separate formations, each sharing the defining number of players on the line of scrimmage for that category, for a total of nine formations used throughout the experiment. On each training trial, the subjects were shown a random formation for 2.5 s and were tasked with pressing a button on a keypad to place the formation into one of the three categories during the 2 s window (Figure 2). Corrective feedback was given to the subject immediately after making their response and it was on the screen for 1.5 s. The inter-trial-interval was optimized for event-related-design while using Optseq2 software and varied between 2 and 8 s [24]. Each formation was shown six times during each training block and there were six total training blocks.

**Figure 3:**
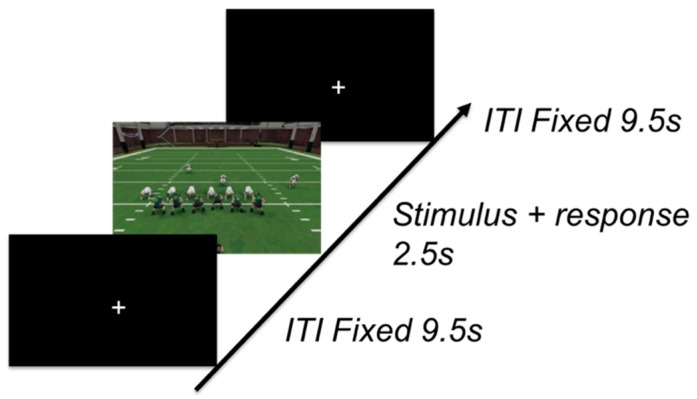
Formations were shown for 2.5 s, regardless of when a subject made a response. No feedback was given. Instead, a fixation cross appeared for a fixed 9.5 s until the next formation was shown.

A generalization block was implemented at the end of the experiment in order to test the subject’s ability to generalize the strategies that they acquired during training. During this block, the nine old formations were intermixed with nine new formations that belonged to the learned category structures. Each stimulus was randomly shown one at a time and was on the screen for 2 s while the subject used a response pad to categorize the stimulus. No feedback was given during this block, and instead a black fixation screen was shown for 10 s before the next stimulus was presented—resulting in a total fixed trial length of 12 s (Figure 3). Each new and old stimulus was shown only once during the generalization block. The inclusion of a generalization block allowed for us to test whether the participants indeed discovered the category structure rather than memorized labels for individual examples, as rote memorization during training would hinder their categorization performance on the new formations.

#### 2.1.3. Procedure

Before coming to the scanning center, the subjects were pre-screened over the telephone to ensure eligibility. Upon arrival at the center, a structural T1 scan was acquired, followed by an exposure block with simultaneous scanning. During this block, the subjects were asked to passively look at the screen and refrain from pressing any buttons. No other context or instructions were given. Each of the nine training formations were shown one at a time for 2 s each before a fixed 10 s ITI. Each formation was shown four times for 36 total trials. Following the exposure block, subjects were read instructions for the experiment. They were told how many formations there would be in the experiment, along with the set number of categories the formations belonged to. Their job was to figure out which formations belong to each category by pressing the buttons on their response pad and utilizing the corrective feedback. A brief practice test (un-scanned) was given, where they learned to categorize unrelated formations. After practice, six training blocks were run with brief breaks in-between and, after training, the subjects sat through another exposure block where they passively viewed each stimulus. To end the experiment, the subjects went through the generalization block, given only the instructions that they were going to go through a final block with no feedback. They were not told whether there would be novel formations in this block. The subjects were asked to write-down their strategies in a debrief questionnaire for categorizing the formations before receiving compensation and leaving the center.

#### 2.1.4. fMRI Acquisition and Pre-Processing

MRI data were acquired with a 3T Siemens Skyra. A high-resolution T1-weighted MPRAGE was acquired for co-registration and normalization before the task was administered (TR = 2.5 s, TE = 3.41 ms, flip angle = 7°, matrix size = 256 × 256, FOV = 256 mm, 1 mm isotropic). Whole-brain fMRI was acquired using a gradient-echo EPI pulse sequence: TR = 2 s, TE = 26 ms, 100 × 100 matrix, FOV = 200 mm, 72 oblique axial slices, no skip, 2 mm isotropic voxels, GRAPA factor 2, multiband factor 3. Preprocessing was carried out in FSL version 5.0.9 (www.fmrib.ox.ac.uk/fsl). The functional images were skull stripped using BET (brain extraction tool), motion corrected, co-registered to the T1 anatomical image, and smoothed with a 4mm FWHM kernel. Two sets of analyses were performed: traditional whole-brain univariate analyses and trial-by-trial multivoxel pattern classification analyses within a set of a priori ROIs. For univariate whole-brain group analyses, functional data were registered to standard MNI space. For multivoxel pattern classification, the data were kept in native space of each participant and trial-specific activation patterns (betaseries) for classification analysis within each participant were extracted using a general linear model with a separate regressor for each trial [25]. A detailed description of each of these analysis approached is provided together with results for better readability.

### 2.2. Results

#### 2.2.1. Behavioral Results

**Figure 4:**
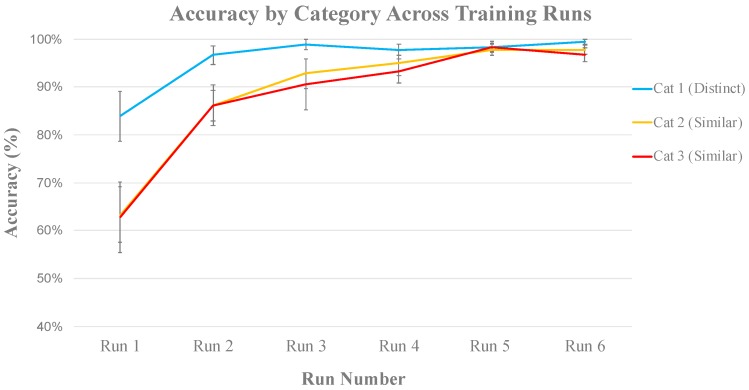
Subjects accurately categorized the visually distinct category quicker than the two visually similar categories. Accuracy for the visually similar categories peaked between runs 4 and 5, which we infer is the time at which subjects discovered the counting rule.

Data from one participant were excluded due to noise caused by motion during scanning, leaving 10 out of the 11 subjects for analysis. Figure 4 shows the average classification accuracy for each category across the six training runs. Visual inspection indicates that participants learned the visually distinct category faster when compared to the two visually similar categories, but by the end of run 4 they were able to accurately identify members of all categories.

**Figure 5:**
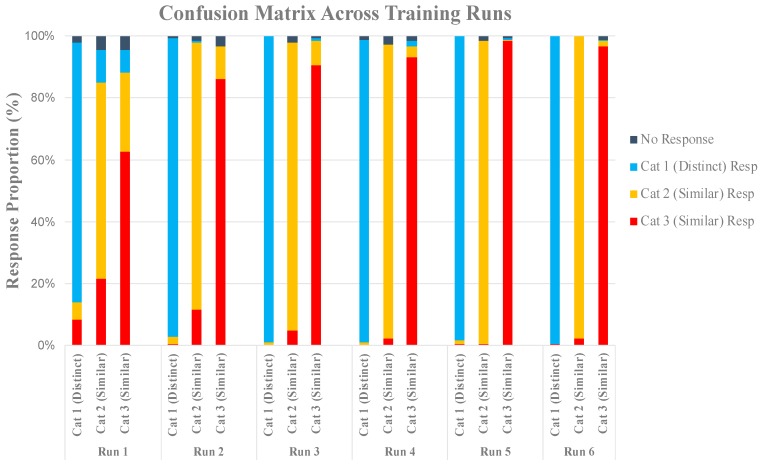
Visualization of the confusion matrix during classification. During the first 3 training blocks, subjects commonly confused the two visually similar categories for one another. By run 4, subjects were able to accurately dissociate between these two categories. Subjects rarely confused any other category when classifying formations in the visually distinct category.

A confusion matrix shows that subjects commonly mixed up the two visually similar categories when making errors, and rarely mixed up the visually distinct category with any other. By block 4, the subjects limited their confusion, which is indexed by the mostly uniformly colored bars in Figure 5. We can infer that this was the point at which most subjects discovered the explicit counting rule that allowed for them to differentiate between members of the two visually similar categories (Figure 5).

**Figure 6:**
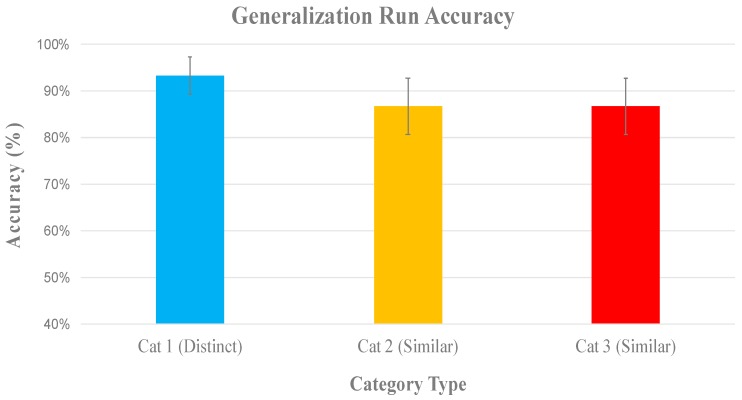
Categorization accuracy in the generalization block.

The generalization run was included to test for the hallmark of category knowledge—the ability to generalize category labels to novel category examples. On average, the subjects completed the generalization run with 92% accuracy for the visually distinct category and 88% accuracy for the visually similar categories (Figure 6). Had subjects been relying on the declarative recall of individual stimuli throughout training, their performance in the generalization run would have been closer to chance (33%).

In the post-test questionnaire, nine out of 10 participants indicated that they used a counting strategy to distinguish between the visually distinct categories, meaning that they accurately identified the defining number of players on the line of scrimmage separating the two categories. The same participants reported relying on perceptual similarity to identify formations in the visually distinct category. This indicates that participants treated the stimuli as expected, using an explicit rule specifically when between-category similarity was high as compared to the within-category similarity. The remaining one participant reported using declarative recall for all stimuli, whereby they memorized each formation individually instead of relying on the intended counting rule. This participant categorized new stimuli in the visually similar categories with an accuracy near chance during the generalization block, which indicated that relying on declarative recall instead of discovering the counting rule was ineffective for generalization in this task.

#### 2.2.2. Univariate fMRI Analysis

##### Training Analysis

**Figure 7:**
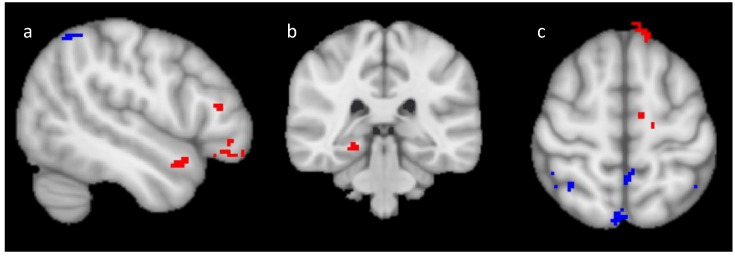
Univariate contrasts of visually similar > visually distinct (Red) and visually distinct > visually similar (Blue) during training displayed in (**a**) sagittal, (**b**) coronal, and (**c**) axial views. Red: dorsal lateral and inferior frontal areas along with hippocampus were engaged significantly more during rule application compared to perceptual similarity analysis. Blue: Fusiform gyrus and lateral occipital cortex were engaged significantly more during perceptual similarity analysis compared to rule application.

Data from each training run and each participant were separately analyzed at a first level analysis using FSL [26]. Visually distinct and visually similar correct trials were separately modeled as two predictors. Each stimulus onset time was convolved with a hemodynamic response function and entered into a general linear model with their temporal derivatives to estimate beta weights. Contrasts of interests were set that tested for differential activation to the two types of categorization trials. Even after performance becomes equated, we focused on data from runs 4, 5, and 6 to explore whether participants engage distinct processes for visually similar vs. visually distinct categories (the runs after subjects could perform the task with proficiency for all three categories: see Figure 4). Data from the three runs within each subject were combined at a second level while using fixed-effects analysis. Data across participants were then combined at the third level using a random-effects analysis (FLAME 1). Figure 7 depicts regions that were more engaged during visually distinct trials over visually similar categorization trials (blue), and vice-versa (red). Individual voxels were considered to be active when reaching a *Z* > 1.8 and surviving a whole-brain cluster size threshold set at *p* < 0.05 [27]. This threshold was used based on the exploratory nature of our small sample pilot experiment, and the reported results were interpreted for the purposes of motivating Experiment 2.

The superior and inferior frontal gyri were engaged significantly more on visually similar categories when compared to the visually distinct category (red clusters, Figure 7). The right hippocampus, a region associated with declarative recall, was also engaged during the classification of visual similar categories, which is consistent with prior work indicating the role of hippocampus in rule-based categorization [28]. During classification to the visually distinct category, the lateral occipital cortex and fusiform gyrus were engaged significantly more as compared to visually similar categories (blue clusters, Figure 7), consistent with what might be expected for similarity-based categorization. Table 1 and Table 2 illustrate a summary of the top 11 regions associated with each condition.

##### Generalization

**Figure 8:**
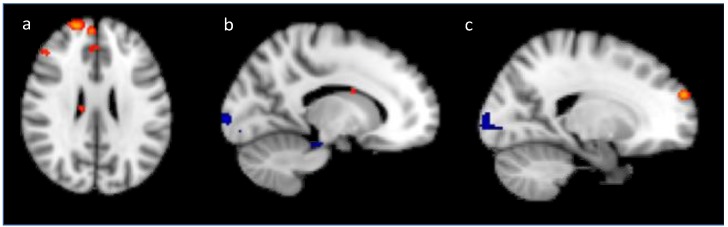
Univariate contrasts of visually similar > visually distinct (Red) and visually distinct > visually similar (Blue) during generalization displayed in (**a**) axial, (**b**) medial sagittal, and (**c**) more lateral sagittal views. Red: Frontal control regions were engaged significantly more during the visually similar trials compared to visually distinct trials during generalization. A cluster over caudate nucleus was also found. Blue: Visually distinct trials relied more heavily on lateral occipital cortex compared to trials separable by a counting rule.

When modeling the generalization block, visually distinct and visually similar trials were further separated, depending on whether they were used during training (old) or whether they were new examples of each category. This resulted in four separate predictors (i.e., Novel-Similar, Novel-Distinct, Old-Similar, Old-Distinct), with the data from each subject being separately analyzed at a first-level analysis. Each stimulus onset time was convolved with a hemodynamic response function and entered into a general linear model with their temporal derivatives to estimate beta weights. Contrast maps were created for each subject, showing areas that were differentially engaged during visually similar vs. visually distinct categories. A group analysis was then run using FLAME 1, combining contrast maps across participants. Figure 8 shows regions that were more engaged during visually distinct trials over visually similar trials (red), and vice-versa (blue). Individual voxels were considered to be active when reaching *Z* > 1.8 and surviving a whole-brain cluster size threshold set at *p* < 0.05 [27].

The results from our univariate analysis show that the left caudate nucleus, left superior frontal gyrus, and left inferior frontal gyrus were significantly more engaged on visually similar trials when compared to visually distinct trials (Figure 8). Caudate nucleus, instead of hippocampus, is one of the only observable differences between the training and generalization contrasts for this condition. Table 3 presents a list of the top 11 clusters from this contrast. In addition, the lateral occipital cortex and right fusiform gyrus were engaged significantly more for distinct trials over visually similar trials during generalization. Table 4 shows a summary of the top 11 clusters.

#### 2.2.3. Multi-Voxel Pattern Analysis

The univariate analyses provided preliminary evidence that participants may engage distinct neurocognitive processes when categorizing visually distinct vs. visually similar trials, albeit at an exploratory threshold. As a second approach, we employed multi-voxel pattern analysis (MVPA; e.g., [29]), which might provide additional sensitivity. Specifically, we asked whether a machine-learning classifier could distinguish, based on the pattern of activation across voxels, into which condition a current trial belonged. First, cortical parcellation and subcortical segmentation was performed while using Freesurfer software for each participant [30,31]. Given prior work on visual memory and the dissociations between rule-based and similarity-based categorization, we focused on regions within the frontoparietal attentional network and the midline, in addition to the posterior visual cortex [32,33,34]. Specific Freesurfer-defined ROIs included superior parietal lobe, anterior cingulate cortex (ACC), medial orbitofrontal cortex (MOFC), inferior parietal lobe, inferior frontal gyrus (IFG), and fusiform gyrus.

One participants’ data were lost between the time we performed the univariate analysis and MVPA due to a site-wide data loss, which left nine out of 11 subjects for MVPA. We modeled the functional data using a separate regressor for each trial to construct a betaseries representing activation patterns that are associated with each individual trial to obtain trial-specific activation patterns for each participant [35]. Each betaseries was smoothed (σ = 3) before being co-registered to each participant’s high-resolution anatomical image while using Advanced Neuroimaging Tools (ANTs) toolbox [36]. Data were kept in native the space of each participant for the classification. Classification analysis used a linear Support Vector Machine (SVM), as implemented by the LinearCSVMC classifier in PyMVPA (pymvpa.org). Data from all runs were included to obtain enough training samples for the classifier. A leave-one-run-out crossvalidation was chosen, as it would maximize the amount of data in each training fold for our sample size [37,38]. Within each ROI separately, the classifier was trained on data from five out of six training runs and tested on the left-out run. The classification accuracy was then averaged across the six cross-validation folds. Two binary (pairwise) classifications were performed: visually similar category 1 vs. visually distinct category, and visually similar category 2 vs. visually distinct category. We subsequently averaged their results together, as there were no differences in classification accuracy between the two (as expected).

**Figure 9:**
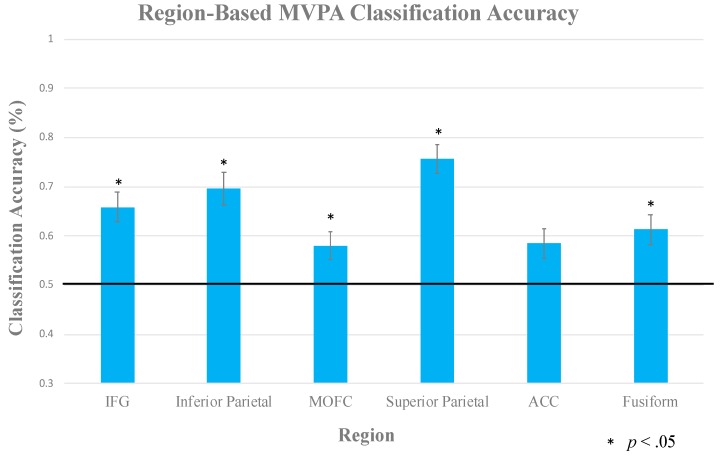
The inferior frontal gyrus (IFG), inferior parietal cortex, medial orbitofrontal cortex (MOFC), superior parietal cortex, and fusiform gyrus were able to classify between our two conditions with significantly above-chance accuracy. Amongst these regions, the superior and inferior parietal cortices provided the most reliable classification. The Anterior Cingulate Cortex (ACC) did not reach statistical significance.

A one-sample *t*-test was used against a baseline value of 0.5 (50% chance for two equally frequent categories). Figure 9 shows the classification analysis in each ROI, indicating dissociable patterns of activation evoked during categorization of stimuli that presumably required rule application vs. those who could be based on perceptual similarity. The IFG (*M* = 0.66; *t*(8) = 4.23, *p* = 0.003), inferior parietal lobe (*M* = 0.70; *t*(8) = 3.65, *p* = 0.007), superior parietal lobe (*M* = 0.76; *t*(8) = 5.8, *p* < 0.001), MOFC (*M* = 0.58; *t*(8) = 3.3, *p* = 0.011), and fusiform gyrus (*M* = 0.62; *t*(8) = 3.75, *p* = 0.006) all predicted category membership with statistically significant accuracy. The ACC (*M* = 0.58) did not reach significance, *t*(8) = 2.02, *p* = 0.078.

## 3. dEEG Experiment

### 3.1. Materials and Methods

#### 3.1.1. Participants

The results from the fMRI pilot motivated the design and interpretation of our fully-powered EEG experiment. Forty-four right-handed participants were recruited from the University of Oregon Human Subjects Pool (22 males, 22 females), with ages ranging between 18 and 39 years old (*M* = 19.5, *SD* = 3.2). All of the participants had normal or corrected-to-normal vision, had no history of head trauma or seizures, and were not consuming medication that could affect their EEG. The participants were pre-screened online for their experience with football to reduce the chance of contextual familiarity confounding differences in skill acquisition rate. Only those subjects that were comfortable recognizing football defensive formations were allowed to participate. The University of Oregon and Brain Electrophysiology Laboratory Company (BELCO) institutional review boards approved the research protocol, and the study took place in the laboratory of BELCO.

#### 3.1.2. Task

**Figure 10:**
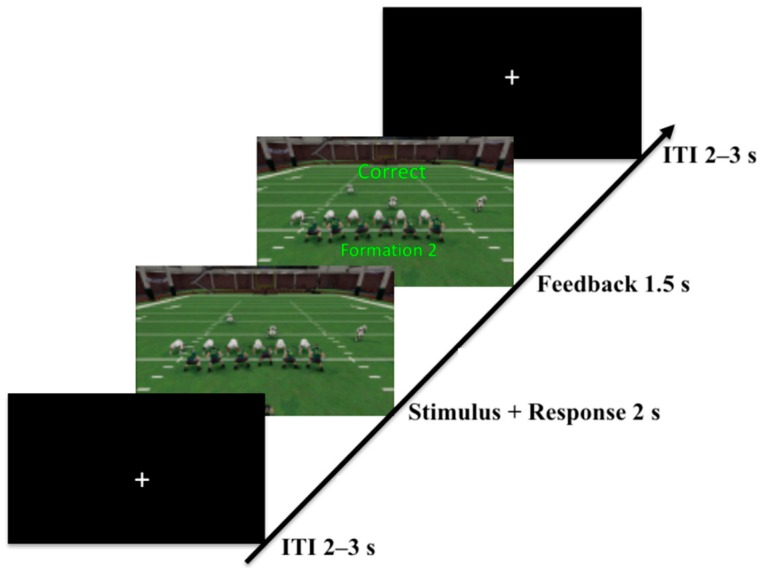
From bottom-left to top right: A fixation cross was shown for 2–3 s. Formations were shown for 2 s while subjects made their response. Immediately following a response, contingent feedback was shown for 1.5 s. Upon feedback termination, a fixation mark was shown for the duration of the inter-trial interval of 2–3 s before the next formation was presented.

The task used in this study was an EEG analogue of the fMRI task used in the pilot. Stimuli in this task consisted of three categories of football defensive formations, with two categories being very visually similar to each other and one category being visually distinct from the other two. For the two similar categories, the subjects needed to discover an explicit counting rule to reliably categorize members of these two groups. For the visually distinct category, subjects could rely on a simple visual similarity analysis to recognize members of this category. Within each category, all of the players were shuffled around the field of view with the exception of the players on the line of scrimmage, as the number of players on the line dictated category membership (Figure 10). This forced subjects to focus their attention to the line of scrimmage over time while ignoring irrelevant players that are positioned elsewhere on the field.

Every category had three formations, each sharing the defining number of players on the line of scrimmage for that category, for a total of nine formations used throughout the experiment. On a given trial, the participants were randomly shown one of the nine formations for 2000 ms and they were instructed to place the stimulus into one of the three categories by pressing a button on a response box within the stimulus exposure window. Once they made a response the stimulus disappeared, and the subject was presented with a corrective feedback screen, which indicated whether they were correct along with text describing the correct category for the stimulus (Figure 10). The feedback was on the screen for 1500 ms, after which a fixation cross with a variable inter-stimulus-interval was shown for 2000–3000 ms. The task was divided into eight training blocks that consisted of 90 trials (or 10 exposures per stimulus) per block, which totaled 80 exposures of every stimulus throughout training.

After the final training block, a generalization block was used, which tested each subject’s ability to apply any rules they developed during training to novel stimuli similar to the fMRI pilot. During the training block, a mixture of the nine training stimuli and nine novel stimuli belonging to the same categories were used. The subjects were not told that the generalization block would include novel stimuli. No feedback was given to the participants after pressing a button to categorize each formation. Instead, a black screen was shown for 1500 ms after a response was made before the fixation cross appeared to begin the next trial. Each old and novel stimulus (18 total stimuli) was shown five times for a total of 90 trials in the generalization block.

#### 3.1.3. Procedure

Following the informed consent process, the participants were fitted with a 256-channel EEG net and placed 55 cm in front of a computer monitor. A chinrest was used to minimize head movements and keep the distance to the monitor fixed for every participant. The participants were explicitly told that there were nine defensive formations in this study belonging to three categories, and that they must learn which formations go into each category. The response feedback that would help teach the participant to make the correct decision was explained clearly, and the participants were allowed to ask questions before the experiment began.

Once the participant could demonstrate an understanding of the study to the research assistant, a short practice block that consisted of 12 trials followed. The formations used in the practice block resembled different basketball formations to avoid familiarity effects once the real training began. After the practice block, eight training blocks occurred, followed by a final generalization block to test a subject’s strategies to novel members of the acquired categories. At the end of the experiment, the participants filled out a debriefing questionnaire, which asked them to describe the strategies that they used to categorize each group of formations. Each session lasted around 2.5 H, and the participants were compensated course credit for their participation.

#### 3.1.4. Learning Criterion

We used the fixed-number of consecutive responses method (FCCR) to simplify the analysis process in order to determine when a participant had sufficiently acquired the response mapping, as we have done in the past [38]. With this method, a subject fulfilled the learning criterion when they could make four correct responses (or non-responses) in a row for each stimulus.

#### 3.1.5. EEG Recording and Pre-Processing

The dEEG was recorded using a 256-channel HydroCel Geodesic Sensor Net (HCGSN) and the data were amplified using a Net Amps 400 Amplifier (Electrical Geodesics, Inc., Eugene, OR). The recordings were referenced to Cz and impedances were maintained below 50 kΩ. dEEG was bandpass filtered (0.1–100 Hz) upon being sampled at 250 s/s with a 16-bit analog-to-digital converter.

After recording, the signals were filtered between 0.1–30 Hz bandpass and segmented into 1200 ms long segments time-locked to the onset of each stimulus (segments extended 200 ms before and 1000 ms after the stimulus onset). Segments containing eyeblinks, muscle tension, major eye movements, or large head movements with 10 or more channels exceeding an absolute voltage threshold of 140 μV were excluded from a participant’s average. Segments containing minor eye movements (saccades) were not fully rejected, given the lack of overlap between the latency and distribution of the saccades with the latency and location of the Medial Frontal Negativity (MFN), LIAN, and P300b (P3b). All of the data were re-referenced to the average reference for analysis.

### 3.2. Results

#### 3.2.1. Behavioral Analysis

**Figure 11:**
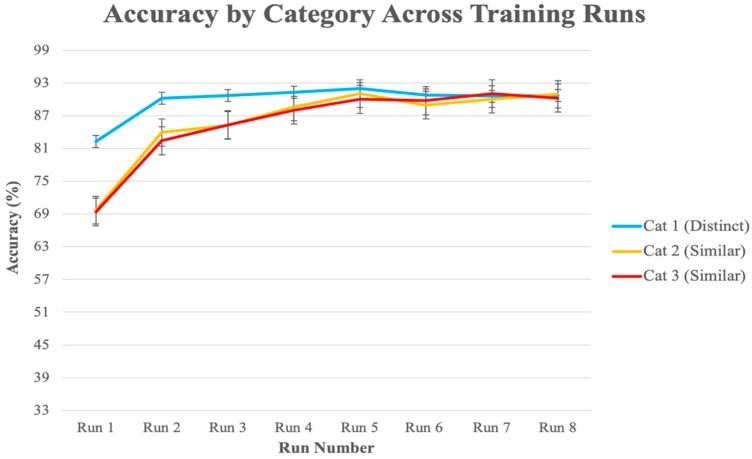
Behavioral performance across training for each category. Participants acquired the visually distinct category first followed by the two visually similar categories. Additionally, there are no behavioral differences between the two visually similar categories. Performance on the two types of categories became equivalent in the second half of training.

Figure 10 shows learning curves for each category. Similar to the behavioral data from our pilot experiment (Figure 4), the participants acquired the visually distinct category first, and there were no performance differences between the two visually similar categories. Based on this, behavioral measures for the two visually distinct categories were averaged together to represent a single visually similar condition in our experiment in order to streamline comparison to the visually distinct category. A paired-samples *t*-test revealed that, on average, across training blocks, the subjects were significantly better at categorizing the visually distinct category (95%) than the visually similar categories (90%), *t*(43) = 5.45, *p* < 0.001. Figure 11 indicates that this difference was driven by early runs, but by Run 5 there were virtually no performance differences across all categories.

Experiment 2 also included a questionnaire that explicitly asked participants to describe the strategies that they used to categorize each formation category. For categorizing visually similar categories, 91% of participants indicated that they used a counting rule when differentiating between the two visually similar categories (e.g., “I counted four players on the line of scrimmage for the first category, and three on for the second category.”), 9% of participants relied on declarative memory for these two categories, and no participant reported reliance on similarity. For categorizing visually distinct formations, 21% of participants reported using an explicit counting rule (e.g., “I counted six people on the line of scrimmage”), 68% reported using declarative recall (e.g., “I memorized each formation individually”), and 11% reported using a perceptual similarity strategy (e.g., “There appeared to be a lot of people on the line of scrimmage for formations in this category, such that I did not need to count any players”). Thus, the self-reported strategies differed between the visually-similar vs. visually-distinct trials, although the distinction was less pronounced than in Experiment 1.

#### 3.2.2. Event-Related Potentials (ERPs) Selection Motivation and Analysis

All EEG data were analyzed using Philips Neuro Net Station 5 software. Classic ERP analysis was chosen, as it allows us to evaluate latency and amplitude differences as a function of categorization strategy. The distinct nature of individual ERPs enables us to attribute any observed differences as occurring within the well-studied circuitry that produces each ERP. In the past we utilized two ERPs to track learning-related changes in the brain: The Medial Frontal Negativity (MFN) and P300b (P3b)—for review see [13,39,40]. The MFN is a stimulus-locked medial frontal component with its primary sources in the Anterior Cingulate Cortex (ACC) [13]. The ACC plays a major role in error monitoring and attention during reward-based learning, which makes it an ideal component for indexing effortful control seen in the early stage of category learning [41]. On the other hand, the P300 is elicited under an array of conditions and there is now a well-defined family of different P300 components. Most relevant to learning, the amplitude of the Late Positive Component (referred to as the P3b) mirrors accuracy improvements during categorization tasks [39,40]. The P3b is hypothesized to reflect a constant monitoring and updating of the context under which learning occurred. As context is formed through learning, the maintenance and updating of the context helps to guide a person toward selecting an action quickly and efficiently. Although the sources of the P3b are still being debated, intracranial EEG and animal studies suggest multiple sources, including Posterior Cingulate Cortex (PCC), medial temporal lobe, and superior temporal sulcus—structures that are integral to the late learning stage [42,43,44,45,46,47,48,49]. In the current experiment, we were interested in examining amplitude and latency differences in these two components as a function of the categorization strategy used on a given trial. In theory, the strategies should differ in their reliance on frontal control areas and posterior corticolimbic structures to complete the task, as seen in Experiment 1. Using ERPs allows for us to interpret latency differences between trial types (distinct vs similar) as reflecting the time-course under which the categorization strategies (and their underlying memory systems) are engaged.

The Lateral Inferior Anterior Negativity (LIAN) is a third component that could potentially dissociate between the two categorization strategies. The LIAN is a lesser-known bilateral component that has shown clear dissociations between the recognition of spatial targets and digit targets in a visuomotor association task [39]. Specifically, the amplitude of the right LIAN is anticorrelated with acquiring the ability to recognize spatial configurations and it shows no changes when targets invoke the phonological loop. However, the amplitude of the left LIAN is positively correlated with learning to recognize phonological targets and it is insensitive to acquiring an ability to perform spatial analyses. The Inferior Frontal Gyrus (IFG) is inferred to be the primary source of these components, but it is worth mentioning that the LIAN is rarely discussed in the literature, where it does not receive any mention outside of its role in visuomotor learning. This component was selected, as it might show a dissociation between the two categorization strategies, since they inherently differ in how they engage the phonological loop.

**Figure 12:**
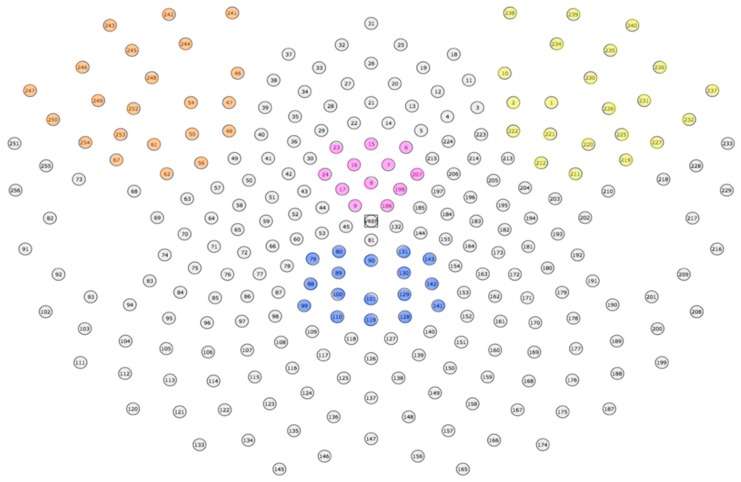
Electrode montages used for the Medial Frontal Negativity (MFN), P300b (P3b), and Lateral Inferior Anterior Negativity Event-Related Potential (LIAN ERP) components. Orange and yellow: Electrodes used for the LIAN analysis. Pink: Electrode cluster used to quantify the MFN. Blue: Electrodes used to quantify the P3b.

**Figure 13:**
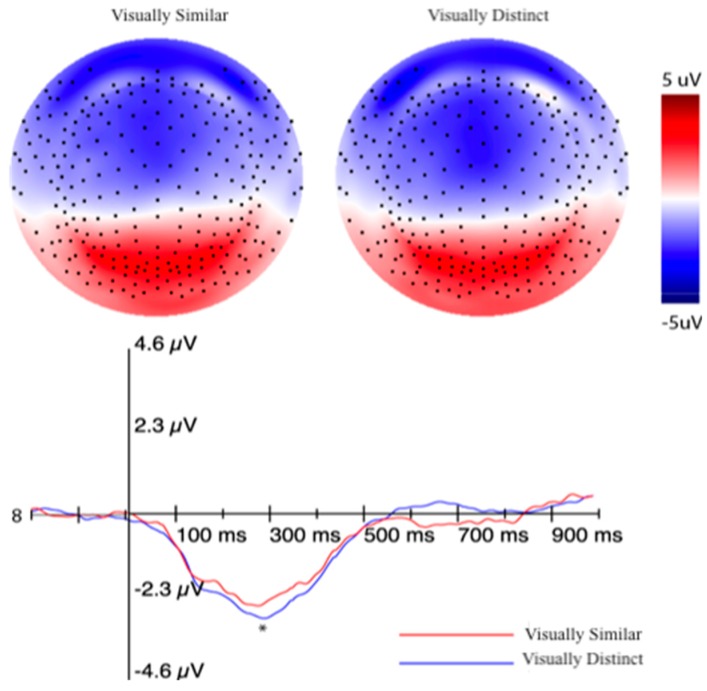
Top: A voltage map displays the voltage across the scalp for the similar and distinct conditions at the peak of the MFN (asterisk in bottom waveform image). A stronger negative voltage is seen over the medial frontal areas for the visually distinct condition. Bottom: Representative waveform (i.e., a single channel over the middle of the negative scalp potential) showing the shape of the MFN for both conditions. This waveform was derived from the grand-average of all the analyzed subjects. The amplitude of the MFN is higher (more negative) for the visually distinct condition.

Please see Section 3.1.5 for a review of how all of the signals were pre-processed. For the MFN analysis, a cluster of 12 electrodes that best represent the medial frontal distribution of the component were chosen (see pink electrodes, Figure 12). Consistent with how we have quantified the MFN in the past, an adaptive mean amplitude corresponding to 20 ms before and 20 ms after the maximum negative peak amplitude in a window that extends from approximately 180–300 ms after stimulus onset was computed for the MFN electrode cluster [13,39]. The MFN was referenced to the preceding positive peak (P200) around 150–200 ms after stimulus onset. This method was applied for the post-learning trials for all three formation categories. The trials in the visually distinct category were averaged together to form a single ERP for the similarity-based condition. After analyzing both visually similar categories individually, we determined that there were no amplitude or latency differences in the MFN between these two categories, consistent with the idea that both would require the engagement of explicit, rule-based categorization. In light of this, trials in the two visually similar categories were averaged together to form a single ERP for the visually similar condition. A paired-samples *t*-test was run to evaluate the differences in MFN amplitude for the visually distinct and visually similar categories. The test revealed a marginally significant effect, such that the MFN was the largest for the visually distinct category (*M* = −2.31 μV) as compared to the visually similar categories (*M* = −2.07 μV), *t*(43) = −1.98, *p* = 0.054 (Figure 13).

**Figure 14:**
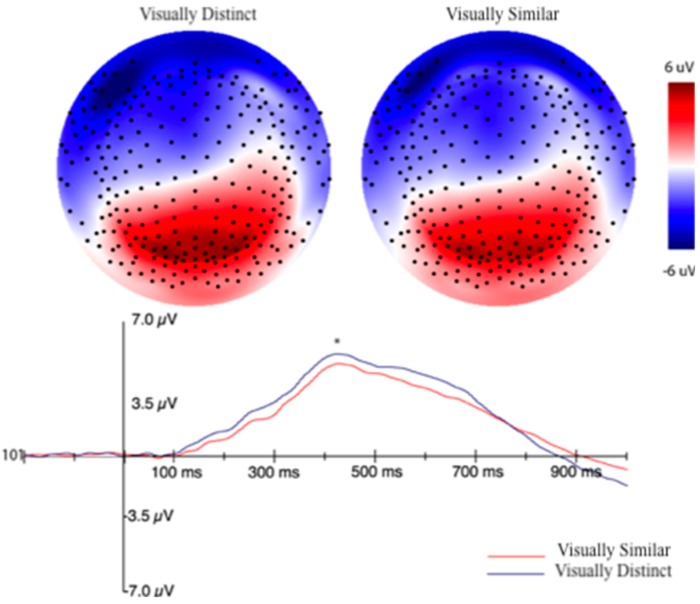
Top: A voltage map displays the voltage across the scalp for the visually similar and distinct conditions at the peak of the P3b (asterisk in bottom waveform image). A stronger positive voltage is seen over the posterior parietal areas for the distinct condition. Bottom: Representative waveform showing the shape of the P3b for both conditions. This waveform was derived from the grand-average of all analyzed subjects. The amplitude of the P3b is higher (more positive) for the visually distinct condition.

For the P3b analysis, a set of 17 channels that corresponded to the posterior-parietal distribution of the component were used (see blue electrodes, Figure 12). An adaptive mean amplitude corresponding to 22 ms before and after the peak amplitude window extending from approximately 450–950 ms after stimulus onset was computed for the group of electrodes to quantify the component. This method was applied for the post-learning trials for all three formation categories and is consistent with how we have quantified the P3b in previous experiments [40]. Separate ERPs were computed for the visually similar and distinct categories similar to the method described for the MFN after establishing that there were no differences in amplitude between visually similar categories. A paired samples *t*-test revealed that the amplitude of the P3b for the distinct category (6.02 μV) was significantly larger than the similar categories (5.34 μV), *t*(43) = 4.17, *p* < 0.001. Figure 14 displays this effect.

**Figure 15:**
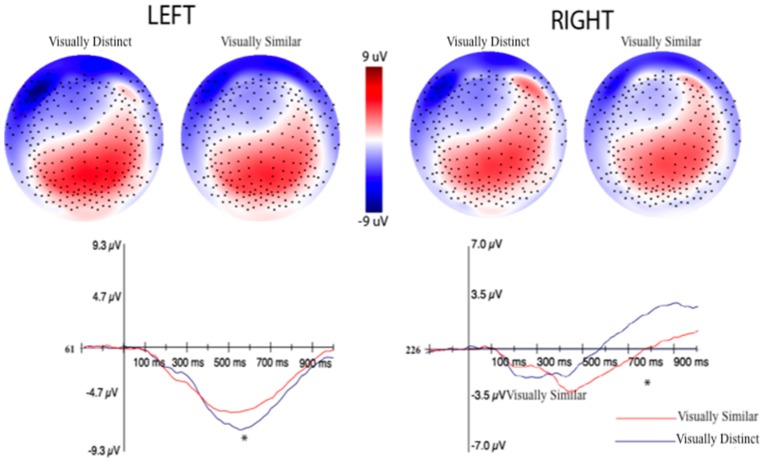
Top: Voltage maps display the voltage across the scalp for the similar and distinct conditions at the peak of the LIAN on the left and right sides (asterisks in bottom waveform images). A stronger negative voltage is seen over the left frontal areas for the visually distinct condition and a stronger negative voltage is seen over the right frontal areas for the visually similar condition. Bottom: Representative waveforms showing the shape of the LIAN for both conditions in the left and right hemispheres. The amplitude of the left LIAN is higher (more negative) for the distinct condition, whereas the right LIAN is higher (more negative) for the similar condition.

The LIAN was quantified by utilizing a cluster of 22 channels in the left or right frontoparietal regions (see orange and yellow electrodes in Figure 12, respectively). An adaptive mean amplitude of these clusters corresponding to 22 ms before and after the peak negative amplitude in a window that extended from 450–950 ms (the same window as the P3b) was used to quantify the component. This method was applied for all post-learning trials for all three categories in each subject. Similar to the P3b and MFN, separate ERPs were computed for the visually similar and distinct categories for both the left and right LIAN after establishing no differences between the visually similar categories. A paired-samples *t*-test showed that the amplitude of the left LIAN was largest for the distinct category (−7.06 μV) as compared to the amplitude of the visually similar categories (−5.54 μV), *t*(43) = −2.98, *p* = 0.004 (Figure 15). However, no significant amplitude difference for the right LIAN were found between the similar categories (−3.55 μV) and distinct category (−2.92 μV), *t*(43) = 1.23, *p* = 0.23.

#### 3.2.3. EEG Machine Learning Analysis

In addition to traditional ERP analysis, we chose to utilize machine learning, as it provides a more data-driven approach to measuring functional differences. We were interested in tracking the earliest timepoint at which brain responses become distinguishable for visually similar vs visually distinct categories. Group-clusters were used to evaluate the general location of these early temporal dissociations. This novel approach has the advantage of utilizing information in the entire pattern of amplitudes across the whole brain, which can potentially increase the sensitivity to subtle differences or engagement of different networks that may include overlapping regions.

For every subject, post-learning trials were chunked into individual segments extending 200 ms before and 1000 ms after stimulus onset for each category. Segments containing ocular or movement artifacts were rejected from analysis. Each segment was baseline corrected while using a 200 ms pre-stimulus baseline before averaging the segments together to form one averaged waveform for each category of stimuli. Waveforms for the two visually similar categories were averaged together to be compared against the distinct category before re-referencing to an average reference. The waveforms were then broken down into their individual samples, which, at a sampling rate of 250 samples/second, resulted in 300 total samples per waveform (each sample representing 4 ms of recording).

**Figure 16:**
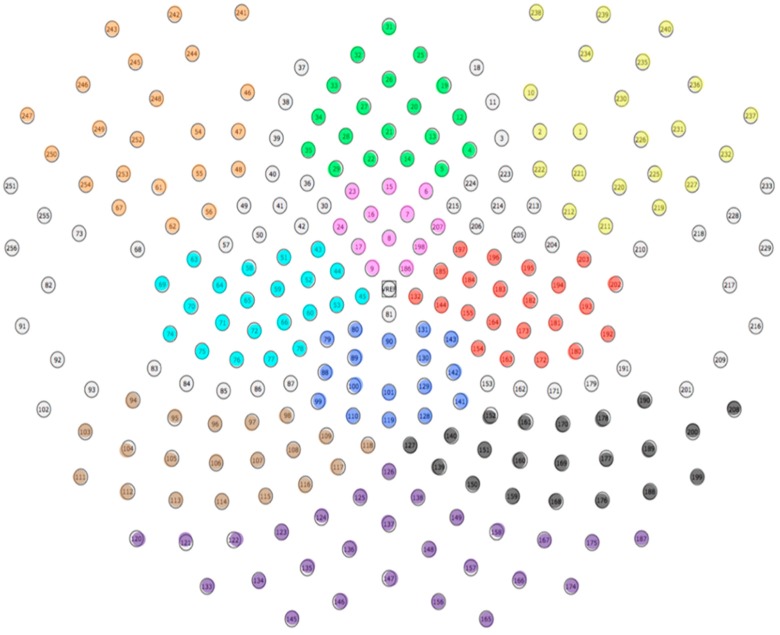
Electrode montages used to define regions during machine-learning analysis. Orange = left frontal, yellow = right frontal, green = medial prefrontal, pink = medial frontal, blue = posterior parietal, cyan = left temporoparietal, red = right temporoparietal, brown = left occipital, purple = medial occipital, and black = right occipital.

We averaged together the raw voltages of electrodes within 10 regions in order to reduce the number of predicting elements in this analysis: left frontal, right frontal, medial prefrontal, medial frontal, posterior parietal, left temporoparietal, right temporoparietal, left occipital, right occipital, and medial occipital (Figure 16). This process was done for each individual sample for both categories. We then averaged together every five consecutive samples together, resulting in 60 timepoints for each waveform with every timepoint representing 20 ms of data. The first 10 timepoints were used in the baseline correction and, thus, not included in the analysis. In the end, this gave us two matrices (one for visually similar and one for visually distinct) for each subject with dimensions 50 (timepoints) × 10 (electrode groups).

For each timepoint, a linear Support Vector Machine (SVM) classifier, as implemented in Matlab, was trained to classify patterns of EEG voltages associated with visually similar vs. visually distinct categories across subjects. The patterns of voltages across all 10 electrode groups associated with each condition for each subject served as the patterns to be classified. Leave-one-subject-out cross validation was carried out, such that patterns from 43 out of the 44 subjects were used to train the classifier, and the subject that was left out of training was used as the test subject. This type of training and test format was iteratively performed until all subjects were used as a test subject. For each iteration and timepoint, the classifier provided an estimate of how likely each of the two test patterns from the left-out subject (one pattern for visually similar trials and one for visually distinct trials) represented the visually similar category. Because there were two categories (distinct vs similar), the classifier-estimated probability that a pattern represents the visually distinct category was always 1 minus visually similar. The test pattern with greater visually similar evidence was labeled as the classifier’s guess for which pattern represents the visually similar category. The other test pattern was labeled as the visually distinct guess. When the classifier’s guess matched the actual condition, the classification was considered correct for the given test participant and timepoint. The classification accuracies from both pairwise classifications (visually similar 1 vs visually distinct, visually similar 2 vs visually distinct) were averaged together. This was done to provide an overall estimate of how well the classifier could distinguish between each of the two visually similar categories vs. the visually distinct category.

**Figure 17:**
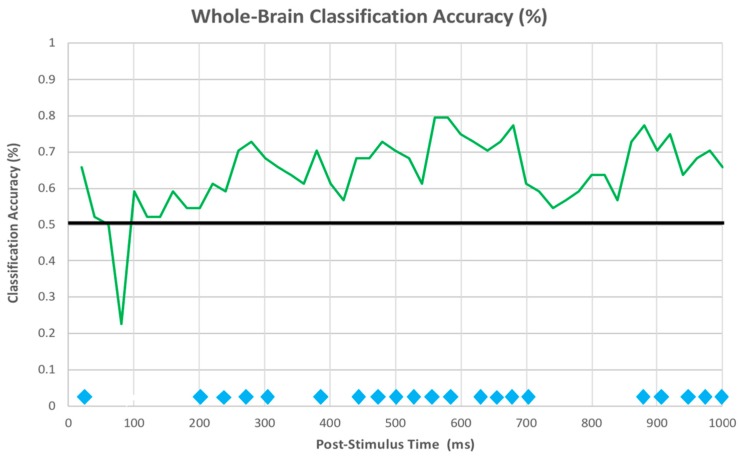
Whole-brain classification accuracy over time on an experimental trial. Blue diamonds along the X-axis represent timepoints where classification accuracy is significantly above chance (*p* < 0.05). The earliest string of above-chance classification accuracies is observable between 200 and 300 ms after stimulus onset, followed by another group between 430–700 ms. A late string of reliable classification occurs around 890–1000 ms.

The classification accuracy for each timepoint was averaged across iterations and a one-sample *t*-test was performed against a theoretical chance mean (50%, as we performed pairwise classifications). The cross-validated classification accuracy for each timepoint is chronologically plotted in Figure 17, and timepoints that had a classification accuracy significantly above chance at *p* < 0.05 (uncorrected) are denoted by a blue diamond along the X axis. From this figure, the earliest timepoints at which the classifier was able to reliable differentiate between the two categories was between 260 and 320 ms, which coincides with the onset and peak of the MFN. Another extended period reliably above chance was between 440 and 700 ms, which corresponded to the peak and onset of the LIAN and P3b.

**Figure 18:**
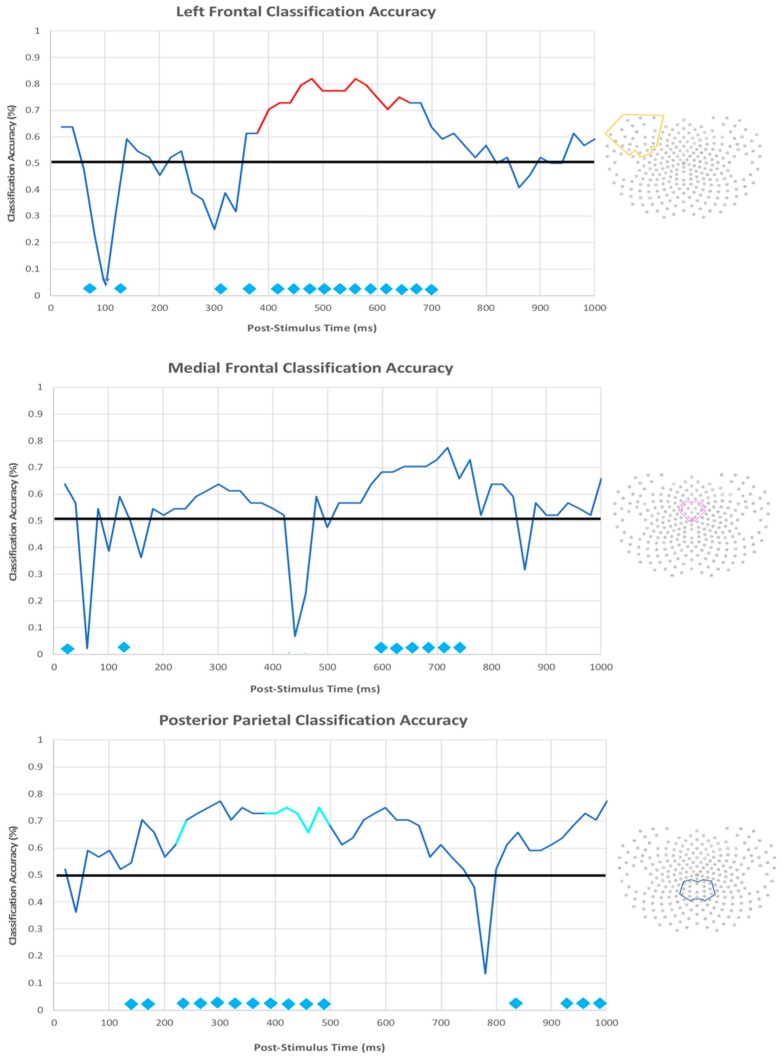
Region-based classification accuracy over time and correlated with behavioral performance. Top: Classification accuracy for the left-frontal electrode montage. Classification accuracy peaks between 400 and 700 ms. During this timeslot, classification is positively correlated with performance. Middle: Classification accuracy for the medial frontal electrode montage. Accuracy peaks between 600 and 750 ms after stimulus onset and does not correlate with behavior in any way. Bottom: Classification accuracy for the posterior parietal electrode montage. Accuracy peaks the earliest in this region, occurring between 220–500 ms. Interestingly, the classification accuracy is negatively correlated with behavioral performance within this window.

The same SVM classification was run again using only the voltages in each region individually to determine whether any one particular region was driving the classification accuracy at each timepoint. The overall classification within each region indicated that the medial prefrontal, left frontal, and posterior parietal regions show the earliest reliable (and strongest) classification accuracy amongst all regions, with a maximum classification accuracy of 82% (Figure 18). Within these regions, reliable differentiation between categories occurs around 250 ms and remains stable until around 740 ms. The classification accuracy peaked earlier in the posterior parietal region compared to the medial prefrontal and left frontal regions, even though we can differentiate between the two categories with reliable accuracy using any of these three regions within the entire 500 ms window.

We were also interested in whether different neural strategies employed for the two types of trials—as evidenced by better SVM differentiation between neural patterns that are associated with each trial type—are beneficial to performance. Thus, we ran an exploratory analysis using a Pearson’s correlation between the SVM classification accuracy and the behavioral performance on the categorization task of each subject. In Figure 18, the trajectory lines are color-coded red or cyan corresponding to timepoints where the SVM classification accuracy was significantly correlated with behavioral performance below a threshold of *p* = 0.05. The timepoints that are highlighted in red indicate that the SVM classification accuracy was positively correlated with behavioral performance, and those in cyan were negatively correlated with performance. The classification accuracy of the medial prefrontal region did not significantly predict behavioral outcome at virtually any timepoint. In contrast, the left frontal region, which is the location of the left LIAN component, was positively correlated with behavior throughout its classification peak. One interpretation of this finding is that the ability to flexibly employ different strategies best matching the current demands may optimize performance overall. Unexpectedly, the classification accuracy of the posterior parietal region was negatively correlated with behavior in several timepoints between 220 and 500 ms. One interpretation of this finding is that the shift away from verbalizable rule-based strategies itself requires executive resources and, thus, excessively differential allocation of resources at this topology and timepoints might make it difficult to continue learning beyond explicit rule-application [50]. Of all the regions, the right frontal area (the location of the right LIAN) was responsible for the very latest classification accuracy peak, occurring between 800 and 1000 ms. The classification accuracy in this region did not significantly correlate with behavior within this window. The three occipital areas along with the two parietal areas failed to demonstrate consistent windows of reliable classification accuracy.

## 4. Discussion

### 4.1. fMRI Pilot Experiment

The main goal of this pilot experiment was to determine the extent to which people engage multiple memory systems during a single categorization task. In line with past literature, the results showed that once subjects acquired the formations in the task, rule-based and perceptual similarity categoriation strategies engaged separate neural systems. These two systems were also recruited during a test block where the subjects were forced to generalize the categorizations strategies that they developed during training. For the machine-learning analysis, regions in the superior and inferior parietal lobes, along with MOFC, fusiform, and IFG successfully dissociated between conditions in the task. This provided enough prelimenary evidence to motivate the fully-powered dEEG experiment.

#### 4.1.1. Univariate Analysis

The categories in this experiment were designed, such that they require the subjects to discover a counting rule to differentiate between two visually similar categories and utilize a perceptual similarity strategy to identify the members of a visually distinct category. Our subjects’ performance on the generalization block supports the assumption that they would recruit the proper strategies. Specifically, they would not have been able to accurately categorize novel formations into the trained categories had they exclusively relied on declarative recall of individual formations.

The superior and inferior frontal gyri were more active during the categorization of visually similar trials when compared to visually distinct trials. These regions are a part of the working memory system, where it is inferred that they are responsible for orienting attention and establishing executive control [22,51,52,53]. In our experiment, the subjects focused their attention toward the players on the line of scrimmage, where they were required to count each player if the formation belonged to one of the two visually similar categories. This is due, in part, to the visually similar categories having low between-category variability, which requires the engagement of a rule-based system. The comparison of the visually distinct category to each of the visually similar categories has much greater between-category variability and, thus, do not require the use of the cognitively taxing rule-based system [54].

Interestingly, caudate nucleus, a region that is integral to rule application, did not reach a level of significance (although this is expected given the small sample size) for the rule-based condition during training. Instead, a cluster over the hippocampus had the highest level of activation during training—a region that is well-known for its role in declarative recall [55]. It is possible that subjects utilized the rule for a short period of time during training, but relied more on the declarative recall of the few relevant players, given that subjects only needed to attend to a single feature within each stimulus to perform categorization (the number of players on the line of scrimmage). However, when encountering novel formations in the generalization block that belong to the categories acquired during training, the subjects were forced into applying the counting rule and, thus, the strong presence of caudate nucleus during generalization could reflect a more consistent reliance on rule application.

In support of our hypothesis, the robust activation of the lateral occipital cortex was present for the visually distinct category when compared to the visually similar categories. This held true throughout training and extended into the generalization block. The lateral occipital cortex has been well-established as the main region governing perceptual similarity categorization [18,19,20,21]. Perceptual similarity categorization can be carried-out with minimal working memory resources and it is optimized for instances with low within-category similarity [21]. The absence of the working memory system when subjects viewed members of the visually distinct category further supports our conclusion that this category engages the perceptual similarity system.

#### 4.1.2. Multi-Voxel Pattern Analysis

Our region-based MVPA showed that the lateral frontal and parietal regions provided the most reliable classification between the visually similar and visually distinct categories, as consistent with previous findings that rule-based categorization requires a higher degree of attentional resources. On the other hand, MVPA provides a more sensitive measure of these conditional effects. More specifically, MVPA provides an avenue for detecting more subtle differences between our conditions that lie within the activity patterns of single regions–information that is sometimes subtracted-out by traditional analyses [56]. These small activation patterns can potentially code for task-relevant information that is important to both memory systems in our experiment.

Frontoparietal regions are well-known for their importance to cognitive control; mainly selective attention to information that is relevant to the task [57,58]. However, non-human primate experiments have demonstrated that activity in the frontal and parietal regions is predictive of an array of different task-relevant features, such as representations of individual stimuli, rule selection, or response selection [59,60,61]. Follow-up studies in humans have shown similar dissociations between stimulus sets and rules using MVPA [62,63]. These task features are essential to the rule- *and* perceptual similarity-based systems. The successful dissociation between category structures while using MVPA over frontoparietal regions in our task supports these previous findings.

### 4.2. Experiment 2 (dEEG)

The primary objectives of this experiment were to further determine whether multiple memory systems are recruited in a single task and evaluate the time course under which these systems are recruited. The results showed that, once the participants acquired the task, clear differences in the Left LIAN, MFN, and P3b components were seen between our two conditions. Overall, the amplitude of each ERP that reflected a difference between the visually similar and visually distinct categories was largest for the similarity-based category. However, the amplitude for the right LIAN was larger for the visually similar categories, although this effect did not reach statistical significance. For the machine learning analysis, the classification accuracy peaked earliest in the posterior parietal region (the location of the P3b), but reliable classification could be performed while using additional electrode clusters, including the left prefrontal and medial prefrontal areas.

#### 4.2.1. ERPs

The MFN amplitude in this experiment was larger for the visually distinct category, albeit the significance of this effect was only marginal. The moderate difference in amplitude between our categories support recent findings that suggest multiple categorization strategies—even those inferred to rely very little on the working memory system—need executive functions in order to select the memory system that is optimal for a task [50]. For stimuli that would benefit most from perceptual similarity, this requirement of effortful control would be very brief—commencing well before an action is committed [50]. The latency of the MFN (180–300 ms) corresponds to the initial orienting of attention in a visuomotor association task and, thus, we propose that the MFN in our task is indexing the controlled attention that is required to select the memory system best suited for categorizing the presented stimulus and does not depend on the optimal system needed to perform a task.

Like the MFN, the P3b in our experiment was larger for the visually distinct stimuli when compared to stimuli that required the application of an explicit rule. Our initial assumption for this component was that the amplitude should be largest for the visually similar categories based on the perceptual similarity literature, which typically describes the robust activation of posterior visual cortex (and no posterior corticolimbic areas) for the visually distinct category [18,19,20]. However, this would only be the case if the participants were exclusively relying on perceptual similarity to categorize members of the visually distinct category. High trial counts could result in subjects utilizing different systems to categorize formations in the visually distinct group as their performance improves. When we analyzed the strategies that subjects were using post-hoc, 89% of participants reported using an explicit counting rule or declarative recall for categorizing the visually distinct formations (68% of the total count being declarative recall and 21% counting rules), while only 11% reported using a perceptual similarity strategy. This theory satisfies the two-stage learning *and* multiple memory systems models, where the early stages of learning are marked by a reliance on a variety of strategies (that may rely on dissociable neural systems) to work toward a more routinized and automatic recall of declarative information. However, more studies are required that track changes in the P3b across training to further associate the amplitude of the P3b with specific categorization strategies. Theoretically, we would see changes in the P3b amplitude as participants progress throughout training and, in turn, that should mirror any changes in the strategy they were using for specific categories.

The amplitude of the left LIAN was the largest for the visually distinct condition, whereas the right LIAN was largest for the visually similar condition, although the latter effect did not reach statistical significance. The left/right conditional flip makes the interpretation of this component fairly difficult. At this time, we are unsure whether both components are interpretable on their own, or if the LIAN is a hemisphere-specific component and the effect observed on the contralateral side is a byproduct of volume conduction. Luu et al. (2007) found that the amplitude of the right LIAN decreased as subjects acquired the ability to perform spatial analyses in a visuomotor association task, but the amplitude of the component remained unchanged when the targets in the task were digits that evoked the phonological loop [39]. They also found that the amplitude of the left LIAN increased as the subjects acquired digit targets in their task, whereas the amplitude remained unchanged as they acquired the ability to perform spatial analyses. Motivated by the findings of their experiment, we drew an initial assumption that the amplitude of both the left and right LIAN should be largest for visually similar condition in the current experiment. As similarly discussed in our interpretation of the P3b, however, this would only be the case if the subjects exclusively relied on perceptual similarity analyses to categorize formations in the visually distinct category—similar to the spatial analyses that were performed in Luu et al. (2007) [39].

Given the vast majority of subjects in our experiment used rote learning to categorize the visually distinct condition instead of the hypothesized perceptual similarity, one interpretation of our findings is to view them as a contrast between declarative recall of individual stimuli (visually distinct category) and explicit rule application (visually similar categories). When viewed from this perspective, the location of the LIAN coincides with structures that are essential for both forms of analysis, such as the temporal lobe and inferior frontal gyrus (IFG) [64,65]. Based on the higher accuracy for the visually distinct category, our findings that the right LIAN was smaller for this category is in-line with meta-analytic findings that show a right hemisphere-specific reduction in anterior temporal and IFG activity with the development of expertise in visuomotor tasks [8]. We could be seeing right hemisphere-specific reductions in the attentional resources that are needed to categorize the visually distinct group of formations simply because our subjects are consistently at a more advanced stage of learning for this condition when compared to the visually similar condition. Our left LIAN results also become more interpretable through this lens. If our subjects are significantly more advanced at declaratively recalling the visually distinct formations, then we would expect the left LIAN to be larger for this condition based on the findings of Luu et al. (2007) [39]. The amplitude of the left LIAN linearly increased for digit targets in their visuomotor learning task, which theoretically engage the same explicit form of memory as both conditions in our experiment. Thus, the left LIAN differences seen in our study could be reflecting differences in expertise between our subject’s ability to categorize the visually similar and visually distinct categories.

#### 4.2.2. dEEG Machine Learning

Using machine learning, we were able to successfully dissociate between our two conditions when utilizing raw voltages distributed across the entire scalp. We were especially interested in the timepoint-by-timepoint classification to identify the earliest point at which we can differentiate between our conditions as subjects view a stimulus. In our study, the onset of a stable period of reliable dissociation was around 200 ms after stimulus onset, which coincides with the initial onset of the MFN ERP component. We interpret this early classification timepoint as reflecting the initial controlled attention required to select a memory system based on the stimulus being presented.

We ran a second machine learning analysis on only the voltages of single groups of electrodes in 20 ms intervals to understand which individual regions were driving the classification accuracy. Our results from this analysis showed that the medial prefrontal, left frontal, and posterior parietal regions collectively contributed to the earliest reliable classification point. fMRI studies using multi-voxel pattern analysis (MVPA) have consistently demonstrated that individual rules can be reliably decoded in frontal and parietal regions [32,33,34]. Our EEG decoding results expand on these findings by specifying that the pattern representations of these concepts coincide with the initial orientation of attention. Through sufficient trial and error learning, the context under which an action is learned in a visuomotor task becomes tied to each individual stimulus in the task [66]. We can assume that the initial conscious registration of a stimulus prompted a conditioned re-establishment of the explicit rules (the learning context) that would dictate their subsequent action selection since we only analyzed trials after our subjects had been sufficiently trained on the task. This theory could explain why the first pattern dissociation between our two categories happens around the earliest time that a person can explicitly orient attention.

### 4.3. Category Learning Strategies as a Function of Expertise

The theories of categorization that formed the basis for our experiments commonly discuss these memory systems individually. However, the results from our experiments indicate that multiple memory systems may develop alongside one another in a single task, alternating from trial-to-trial to meet task demands. The development of expertise within each system could happen independently, and they likely share the same end-goal of automating the attention process with extended training.

Palmeri (1997) made one of the first attempts at describing the time that it takes subjects to reach automaticity while using perceptual similarity versus rule-based categorization [67]. In one experiment, Palmeri had subjects categorize objects with high within-category similarity, whereas in a separate experiment had subjects categorize objects with high between-category similarity, which required the discovery of a rule. The results from these experiments demonstrated that subjects utilizing perceptual similarity reached automaticity notably faster than those that relied on rules. This led to the development of a new theory termed Exemplar-Based Random Walk (EBRW) which proposes that, when a probe is presented, exemplars stored in memory race to be retrieved with a speed that is proportional to their similarity to the probe. Each one of the retrieved exemplars drives a random walk until sufficient evidence is presented. Once enough evidence has been retrieved, a subject makes a response [68,69].

Computational models of EBRW allow for the reaction times to be sped up by increasing within-category similarity and increasing the number of exposures to an exemplar [67]. This would result in a shorter training period before subjects reach automaticity when categorizing visually similar exemplars. The model also accounts for a longer training period when the subjects are forced to rely more on the random walks or the evidence-gathering aspect of the process when the categories have low within-category similarity and/or high between-category similarity, which was the case for our visually similar categories. EBRW, when interpreted on a conceptual level, helps to explain how implicit and explicit forms of categorization are a simple function of expertise development. The different strategies are called upon, depending on the structure of a category being presented and they share the common function of serving as an intermediate strategy before transitioning to an automatic mode of operation. However, a potential shortcoming of EBRW is that it postulates a single, unitary memory system underlying performance, which does not align well with neuroscience evidence in favor of multiple category learning systems [15,70]. We propose that this theory be altered to accept these processes as the work of distinct memory systems. It is clear that future work is needed to develop new theories for how these distinct systems develop under learning conditions that may require more than one type of system to optimize performance.

### 4.4. Alternative Interpretations and Limitations

While we interpret the neurophysiological differences between categories to reflect the use of different categorization strategies, a key challenge to clear interpretation is that the conditions differ in difficulty. The subjects had an easier time recognizing and categorizing the visually distinct category, whereas it took longer to do the same for the visually similar categories. The current task and result can be alternatively framed in terms of the differences in the relative contribution of top down vs. bottom up processes during learning. Specifically, for the visually distinct categories, subjects could largely rely on bottom-up (stimulus-driven) signals. In contrast, the categorization of visually similar categories requires a greater involvement of top-down signal guiding attention to relevant details to implement an explicit counting rule. Relatedly, we can view our results from a general cognitive resources framework. As stated earlier, the two visually similar categories have a relatively small between-category variance, which would require more working memory resources to discern, and arguably engage, the rule-based categorization system. On the other hand, the between-category variance between each of the two visually similar categories as compared with the visually distinct category is much higher. In theory, making a distinction with high between-category variance should not be as cognitively taxing. The differences seen in our ERPs reflect the differential allocation of cognitive resources, and this difference has been argued to be controlled by dissociable memory systems [15,54].

Unfortunatley, a fundamental feature of naturalistic learning environments is that some deviation in individual learning strategy is expected. Although we make the argument that rule-based and perceptual simliarity-based judgements play an intermediate role on the path to declaritive recall and automazation, there is no clear way to determine whether the subjects switched their strategies with extended training in the current experiments. A future experiment is necessary to further explore the finer details of any inferred stategy shifts related to expertise.

## 5. Conclusions

A large number of studies have outlined the behavioral and neural processes that are associated with different methods of categorization. The overwhelming consensus amongst these studies is that different categorization strategies serve the purpose of making learning as efficient as possible under different learning conditions. These strategies rely on distinct memory systems. A common feature of category learning studies is that they use tasks that are designed to recruit these systems and strategies one at a time. Yet, real-world learning likely involves the ability to switch between memory systems, including different approaches to different stimuli within a seeming same task. Through the conducted experiment, we provided initial evidence that people can switch between memory systems to optimize performance in a single task. In addition, we determined the time course by which the brain shows dissociable neural signatures signifying the selection of these different memory systems.

## Figures and Tables

**Table 1:** Cluster location and size for similar > distinct contrast in blocks 4, 5, and 6.

Location	Cluster Size	*Z*-Value	X	Y	Z
L Sup. Fr. Gyrus	58	2.79	−54	44	−10
L. IFG	50	2.95	−50	30	14
L. Sup. Fr. Gyrus	38	2.72	−12	40	56
L. Sup. Fr. Gyrus	34	2.47	−16	56	38
R. Hippocampus	26	2.88	22	−34	−10
L. Sup. Temp. Gyrus	25	2.67	−50	10	−16
R. Fusiform Gyrus	25	3.04	40	−44	−20
R. Lateral Occipital Cortex	24	2.72	−10	−12	56
L. Suppl. Motor Cortex	22	2.42	58	−64	24
Brain Stem	22	2.63	6	−22	−28
R. Mid. Temp. Gyrus	20	2.56	40	−58	2

**Table 2:** Cluster location and size for distinct > similar contrast in blocks 4, 5, and 6.

Location	Cluster Size	*Z*-Value	X	Y	Z
R. Lateral Occipital Cortex	519	3.16	6	−74	36
R. Lateral Occipital Cortex	154	2.87	34	−62	62
L. Fusiform Gyrus	106	3.17	−20	−66	−18
L. Lateral Occipital Cortex	98	2.83	−36	−56	38
R. IFG	89	3.25	20	56	−6
L. Post. Cingulate Gyrus	70	2.88	−8	−40	48
R. Lateral Occipital Cortex	55	2.47	20	−88	38
R. Fusiform Gyrus	52	2.62	20	−54	−16
L. Middle Frontal Gyrus	49	2.77	−38	45	18
R. Occipital Pole	41	2.34	20	−104	−10
Brain Stem	40	3.05	22	−32	−42

**Table 3:** Cluster location and size for similar > distinct contrast in generalization block.

Location	Cluster Size	*Z*-Value	X	Y	Z
L. Caudate Nucleus	290	3.5	−8	−10	24
Cerebellum	129	3.22	16	−72	−28
Cerebellum	125	3.43	32	−80	−22
Cerebellum	90	3.18	4	−50	−10
L. Sup. Frontal Gyrus	88	3.21	−28	6	64
L. Lateral Occipital Cortex	73	3.17	−26	−78	50
R. Lateral Occipital Cortex	71	3.08	40	−74	42
L. Inf. Frontal Gyrus	67	3.22	−42	22	4
Cerebellum	63	3.3	−26	−90	−26
L. Sup. Frontal Gyrus	59	3.17	−42	46	20
Brain Stem	58	2.92	14	−16	−38

**Table 4:** Cluster location and size for distinct > similar in generalization block.

Location	Cluster Size	*Z*-Value	X	Y	Z
R. Lateral Occipital Cortex	1922	4	18	−100	6
R. Fusiform Gyrus	335	3.41	12	−72	−2
R. Inf. Frontal Gyrus	213	3.11	62	6	12
Postcentral Gyrus	144	3.35	−40	−26	54
L. Sup. Temporal Gyrus	143	3.49	68	−24	28
Cerebellum	113	3.09	−20	−72	−52
L. Fusiform Gyrus	100	2.88	38	−54	−24
R. Mid. Temporal Gyrus	99	3.13	66	−40	2
R. Mid. Frontal Gyrus	82	3.25	32	18	30
R. Mid Temporal Gyrus	79	3.37	54	−6	−28
R. Angular Gyrus	70	3.7	56	−46	30
